# The Late Dr. Andrew Combe

**Published:** 1847-10

**Authors:** 


### THE LATE DR. ANDREW COMBE.

[We are indebted to the columns of that very superior newspaper, The Scotsman, for the following excellent account of an excellent man—if ever such there was. We have reason to believe that it is from the pen of a celebrated writer as well as a kindred spirit, who knew the deceased long and well—Mr. Robert Chambers. In all that is therein said in commendation of the character of Dr. Combe, we so entirely concur—and we speak from long personal intercourse—that if we could wish any of the expressions altered, it would be only that they might be made still stronger and more emphatic. Never, we will venture to say, did the ranks of Physic lose a more estimable member; and rarely—very rarely—has the grave closed over a gentler, truer, wiser, or better man. His loss to his friends is a loss that can never be supplied; his loss to the community is one of the greatest it could sustain in losing an individual. But he had fulfilled his mission, and done his work as far as was permitted: may they who are left to lament him, strive, as far as in them lies, to emulate his bright example.]

Dr. Combe's death took place at Gorgie Mill, near Edinburgh, on Monday, the 9th of August, when he had nearly attained the age of fifty years. Since 1820 he had laboured under severe pulmonary consumption,* which frequently interrupted his practice, compelled him to spend many winters in France, Italy, or Madeira, and at length, by wholly unfitting him for the active duties of his profession, gave him that leisure which he turned to so excellent account in the preparation of his well-known works on Health and Education. In April last, hoping to receive benefit from a voyage, he paid a short visit to the United States; and although this hope was disappointed, his health did not appear to have suffered from the exertion, and it was not till within a few days of his death that his condition became alarming. The immediate cause of that event was chronic disease of the bowels, which suddenly came to a crisis, and was such as to defy every effort of medical skill. His sufferings were extremely slight, and he displayed to the end that cheerfulness and serenity which was a prominent feature of his character during life.

Dr. Combe was born at Livingston's Yards, a suburb under the south-west angle of the rock of Edinburgh Castle, on the 27th of October, 1797. His father—who carried on the business of a brewer, and was remarkable for his worth and unassuming manners—had married in 1775 a daughter of Abram Newton, Esq., of Curriehill, noted among her acquaintances for her skilful domestic management and general activity and good sense. From this union sprang a family of seventeen children, among whom Andrew held the place of fifteenth child and seventh son. In those days, the district we have named abounded still more than it does at present with offensive pools and ditches, the noxious influence of which, in conjunction with defective ventilation of small and crowded sleeping-apartments at home, appears to have been a chief cause of the disease and early mortality

* The examination after death showed great disease of one lung, but the affection was not, strictly speaking, *consumption*, as there were no tubercles there or elsewhere.—Ed.

which prevailed in the family. Certain it is that his youthful experience and observation supplied Dr. Combe with not a few of the examples by which he afterwards impressed on his readers the evil effects of ignorance of the laws of the human constitution.

Having gone through the usual course of instruction at the High School, he was bound apprentice to the late Henry Johnston, Esq., surgeon in Edinburgh; and in 1817 had acquired an amount of skill and knowledge which enabled him then to take a surgeon's degree. With the view of farther qualifying himself for medical practice, he next repaired to Paris, where two years were laboriously spent under the tuition of such men as Dupuytren, Esquirol, and Spurzheim. In 1823 he began to practise in Edinburgh, and about two years later took there his medical degree. The conscientiousness, kindliness, and sagacity which he displayed as a physician, and the extensive knowledge he had acquired of his profession, speedily brought him a flourishing practice, which became every year more extensive, till a return of pulmonary symptoms obliged him in 1831 to proceed once more to Italy. He was, however, able to pass the winter 1832-3 in Scotland, and in the latter year to resume his practice. In 1836 he was honoured with the appointment of Physician in Ordinary to the King and Queen of the Belgians, and for several months attended the royal family in Brussels; but the climate proving unfavorable to him, an alarming return of the pulmonary symptoms abruptly sent him back to recruit his health in his native land. Subsequently he continued to act as Consulting Physician to their Majesties, and occasionally paid them a visit. About six or seven years ago he was appointed one of the Physicians Extraordinary to the Queen in Scotland, and afterwards one of her Majesty's Physicians in Ordinary in this part of the United Kingdom. He was also a Fellow of the Royal College of Physicians of Edinburgh, and a Corresponding Member of the Imperial and Royal Society of Physicians of Vienna.

The works by which the name of Dr. Combe is best known to the public are—'The Principles of Physiology applied to the Preservation of Health, and to the Improvement of Physical and Mental Education,' of which twelve editions have been called for since its first appearance in 1834; 'The Physiology of Digestion considered with Relation to the Principles of Dietetics,' originally published in 1836, and now in the seventh edition; and 'A Treatise on the Physiological and Moral Management of Infancy, for the use of Parents,' of which the first edition came out in 1840, and the fifth in the present year. In preparing these works, his constant aim was to exhibit the relation subsisting between the rules of conduct recommended and the particular laws of the organization according to which their influence is exerted, so that the recommendation might rest, as far as possible, on the foundation of Nature, and not on his mere personal authority. He wished to make his readers understand *why* certain courses are beneficial and others hurtful—to exhibit *principles* in a clear and intelligible manner, so that every individual might be enabled to adapt his conduct rationally to his own peculiar circumstances. He urges, that as every organ of the body has a specific constitution, and is regulated in its action by fixed laws appointed by Divine Wisdom, success in avoiding causes of disease, and in removing them when they come into play, will greatly depend on the extent of our knowledge of the nature and laws of the various organs, and their relations to each other and to external objects. "In teaching dietetic rules and hygienic observances, therefore," says he, "the precepts delivered should be connected with and supported by constant reference to the physiological laws from which they are deduced. *Thus viewed, they come before the mind of the reader as the mandates of the Creator;* and experienee will soon prove that by his appointment, health and enjoyment flow from obedience, and sickness and suffering from neglect and infringement of them." The words we have put in italics express an idea on which he frequently dwells with earnestness in his works, and which he delighted in private conversation to enforce. The extent to which these valuable treatises have been circulated, and not merely read but *studied,* shows that he did not mistake the manner in which such instruction can be successfully imparted.

In 1820 was instituted the Phrenological Society, of which he and his elder brother, Mr. George Combe, were among the leading members. He contributed two essays to the volume of Transactions published by that body in 1824, and subsequently wrote many valuable papers in the *Phrenological Journal,* which was commenced in 1823, and now extends to twenty volumes. In 1831 he published 'Observations on Mental Derangement; being an Application of the Principles of Phrenology to the Elucidation of the Causes, Symptoms, Nature, and Treatment of Insanity.' This work has long been out of print, and is thus referred to in the preface to the eleventh edition of his 'Physiology applied to Health and Education:' "As many inquiries continue to be made for a new edition of my 'Observations on Mental Derangement,' I avail myself of this opportunity to state that infirm health having prevented me from devoting much attention to the treatment of insanity for some years past, and consequently disqualified me from doing that justice to the subject which its later progress and inherent importance imperatively demand, I have, although with great reluctance, abandoned all present intention of reprinting the work." In the beginning of 1846, his strong conviction of the importance of phrenology to medical men induced him to write, at the expense of considerable fatigue, an 'Address to the Students of Anderson's University, Glasgow, at the opening of Dr. Weir's first course of Lectures on Phrenology.' It was delivered to a crowded audience by his brother, and subsequently appeared as a pamphlet. He also contributed several articles to the *British and Foreign Medical Review,* and was an occasional writer on medical and sanitary subjects in our own columns.

The decease of Dr. Combe will have taken no one who knew him by surprise, for he was for many years in that condition which makes life a greater miracle than death; but it will not on this account be the less deplored, either as causing a blank in the circle of private friendship, or as the signification of a public loss. Dr. Combe belonged to that rare class of physicians who present professional knowledge in connexion with the powers of a philosophical intellect, and yet in practical matters appear constantly under the guidance of a rich natural sagacity. All of his works are marked by a peculiar earnestness, lucidity, and simplicity, characteristic of their author; they present hygienic principles with a clearness for which we know no parallel in medical literature. To this must be ascribed much of the extraordinary success they have met with, and on this quality undoubtedly rests no small portion of their universally acknowledged utility. Those, however, who look below the surface will not fail to trace a deep philosophical spirit as pervading these works, something arising from a perfect apprehension of, and a perfect allegiance to, the natural rule of God in our being. It has been a guidance—we would almost say an inspiration, to the author, without ever carrying him for a moment where ordinary readers could not follow him. Here we think is the true though latent strength of Dr. Combe's popular writings, and that which will probably give them a long-enduring pre-eminence in their particular department. We always feel, in reading them, that we are listening to one of those whom Nature has appointed to expound and declare her mysteries for the edification of her multitudinous family. In his own section of her priesthood, certainly few have stood in his grade, fewer still become his superiors.

The personal character and private life of Dr. Combe formed a beautiful and harmonious commentary upon his writings. In the bosom of his family and the limited social circle to which his weakly health confined him, he was the same benignant and gentle being whom the world finds addressing it in these compositions. The same clear sagacious intelligence, the same entire right-mindedness, shone in his conversation. An answer to any query put to him, whether respecting professional or miscellaneous matters, was precisely like a passage of one of his books, earnest, direct, and conclusive. Whatever, moreover, he called upon others to do or to avoid, that he did and that he avoided, in his own course of life; for doctrine with him was not something to be treated as external to himself, but as the expression of a system of divine appointment of which he was a part. To his rigid, though unostentatious adherence to the natural laws which he explained, it was owing that he sustained himself for many years in a certain measure of health and exemption from suffering, while labouring under the consumptive tendency which finally has cut short his career. On this point there is the more reason to speak emphatically, when we reflect that the years thus redeemed from the grave were employed in that which will yet save many from premature death, as if it had been his aim to show the value of even the smallest remains of life and strength, and thus advance one of the principles dearest to humanity. It was not, however, in any of these respects that the character of Dr. Combe made its best impression, but in his perfect geniality and simplicity, and the untiring energy of his practical benevolence. Here resided the true charm of his nature, and that which made him the beloved of all who knew him. No irritability attended his infirm health; no jealousy did he feel regarding those whom superior strength enabled to outstrip him in the professional race. Kindly and cordial to all, he did not seem to feel as if he could have an enemy—and therefore, we believe, he never had one. It might almost have been said that he was *too* gentle and unobtrusive—and so his friends, perhaps, would have thought him, had it not on the other hand appeared as the most befitting character of one who, they all knew, was not long to be spared to them, and on whom the hues of a brighter and more angelic being seemed already to be shed.

